# Psychiatric disorders associated with PCSK9 inhibitors: A real‐world, pharmacovigilance study

**DOI:** 10.1111/cns.14522

**Published:** 2023-11-10

**Authors:** Zhifang Deng, Jue Liu, Hongjian Gong, Xiaonan Cai, Han Xiao, Wenqi Gao

**Affiliations:** ^1^ Department of Pharmacy, The Central Hospital of Wuhan Tongji Medical College, Huazhong University of Science and Technology Wuhan China; ^2^ Institute of Maternal and Child Health, Wuhan Children's Hospital (Wuhan Maternal and Child Healthcare Hospital) Tongji Medical College, Huazhong University and Technology Wuhan China

**Keywords:** FDA adverse event reporting system, PCSK9i, pharmacovigilance, psychiatric disorders

## Abstract

**Background:**

The relationship between Protein Convertase Subtilisin Kexin Type 9 inhibitor (PCSK9i) and psychiatric adverse events (AEs) remains unclear due to the limitations of clinical trials. In this study, PCSK9i‐related psychiatric AEs were realistically observed and systematically summarized in the real world by data mining the FDA AE Reporting System (FAERS).

**Method:**

Total AEs between the third quarter of 2015 and the first quarter of 2023 were obtained from FAERS. Psychiatric AEs were identified using disproportionality analysis and clinical prioritization of signals using a rating scale, followed by univariate logistic regression to explore factors influencing psychiatric AEs.

**Results:**

Psychiatric AEs accounted for 6.7% of the total number of PCSK9i reports. Eighteen psychiatric AEs were defined as PCSK9i‐related psychiatric adverse events (ppAEs) (lower 95% CI of both ROR >1 and IC_025_ > 0). The median age of ppAE reports was 68 years, and female patients accounted for 22.67% of reports, including 41.40% of reports with a serious outcome. Eleven (61.11%) and seven (38.89%) ppAEs were classified as weak and moderate clinical priority, respectively. The median time to onset of ppAEs was 149 and 196 days after treatment with evolocumab and alirocumab, respectively. Patients weighing ≥80 kg were 1.59 times more likely to experience ppAEs.

**Conclusion:**

The results of this study facilitate the prioritization of psychiatric AE signals by healthcare professionals with the goal of mitigating the risk of PCSK9i‐related psychiatric AEs. However, as an exploratory study, our findings need to be confirmed in large‐scale prospective studies.

## Graphical abstract

This pharmacovigilance study is based on real‐world data from the FAERS database. Total AEs between the third quarter of 2015 and the first quarter of 2023 were obtained from FAERS. Psychiatric AEs were identified using disproportionality analysis and clinical prioritization of signals using a rating scale, followed by univariate logistic regression to explore factors influencing psychiatric AEs.

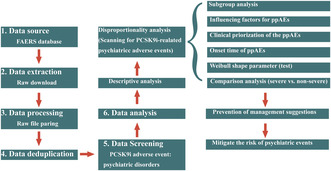

## 1 INTRODUCTION

Lipid‐lowering drugs are among the most commonly prescribed medications worldwide. A new class of drugs called Protein Convertase Subtilisin Kexin Type 9 inhibitor (PCSK9i) has been approved by the U.S. Food and Drug Administration (FDA) and the European Medicines Agency (EMA) in 2015 for the treatment of primary hyperlipidemia and familial high cholesterolemia. PCSK9 induces hypercholesterolemia by increasing the rate of LDL receptor degradation, preventing diffusion of LDL from plasma into cells, assisting LDL‐bound cholesterol in plasma, and inhibiting the metabolism of LDL cholesterol (LDL‐C).^1^ Evolocumab (IgG2 isotype) and alirocumab (IgG1 isotype) are two types of PCSK9i drugs that are injectable monoclonal antibodies.^2^ They reduce LDL‐C levels by 50%–60% by binding to PCSK9 in the circulation, preventing it from binding to LDLR and reducing LDLR degradation.^3^

Although PCSK9i has been widely used in lipid‐lowering therapies and clinical trial evidence suggests that it appears to be well tolerated, the number of pharmacovigilance studies on PCSK9i is increasing. Neuropsychiatric disorders, one of the adverse event (AE) categories of PCSK9i, have a low incidence and vary in severity of symptoms. In 2012, the FDA warned that cholesterol‐lowering medications may cause serious and reversible cognitive side effects such as memory loss, amnesia, and confusional state.^4^, ^5^

Given the very low LDL‐C level induced by PCSK9i and the importance of cholesterol for brain function,^6^, ^7^, ^8^ the FDA directed PCSK9i developers in early 2014 to focus on monitoring for neurological AEs.^9^ Surprisingly, past studies are not in agreement as to whether PCSK9i impairs neurologic function. Several clinical trials and post‐marketing studies have reported neurologic AEs with PCSK9i. Early Phase 2 safety studies reported a tendency for PCSK9i to trigger cognitive impairment.^7^ The OSLER (Open‐Label Study of Long‐Term Evaluation against LDL‐C) study found that 0.9% of patients treated with evolocumab experienced cognitive AEs, including delirium, attention deficit, dementia, perceptual deficits, and psychiatric disorders, compared with 0.3% of patients in the standard of care group.^10^ Data from the ODYSSEY LONG TERM trial showed that 1.2% of patients in the alirocumab group developed memory impairment compared to 0.5% in the placebo group.^11^ In an analysis of Eudravigilance, Mauro et al. found that 22.7% of reports with PCSK9i described psychiatric AEs.^12^ However, a meta‐analysis based on 39 clinical trials showed that the use of two PCSK9i drugs was not associated with an increased risk of cognitive AEs.^13^ Giugliano et al. followed 1204 patients (mean age 65 years) treated with evolocumab for 1.6 years and found no association with cognitive AEs.^14^ Because of the inconsistent results of previous studies and the paucity of real‐world evidence on age and sex differences, there is a strong need for more reports on the long‐term effects of PCSK9i, especially long‐term safety data from post‐marketing surveillance, which can help to develop appropriate, targeted, and safe therapeutic strategies for patients treated with PCSK9i. The U.S. FDA AE Reporting System (FAERS) is a publicly available database of safety reports submitted by physicians, nurses, patients, and pharmaceutical companies that contains real‐world reports of AEs from a wide variety of populations that may have been overlooked in carefully designed clinical trials.^15^ In recent years, FAERS has played an important role in identifying new, rare, and serious AEs and has become a very valuable resource to support post‐marketing surveillance and pharmacovigilance studies.^16^, ^17^ The aim of this study was to provide an in‐depth and comprehensive understanding of PCSK9i‐related psychiatric AEs using standardized information in FAERS and to describe the clinical characteristics of psychiatric AEs in terms of disproportionality analysis, stratified analysis, clinical priority of signals, time of occurrence, serious outcomes, and contributing factors to provide a useful reference for clinical practice.

## 2 METHOD

### 2.1 Study design

Ongoing post‐marketing surveillance of adverse reactions is imminent due to the limitations of clinical trials, such as relatively small sample sizes, limited duration of follow‐up, and stringent inclusion, and exclusion criteria. FAERS plays an important role in detecting and identifying new, rare, and serious adverse drug reactions and events. The FAERS database contains real‐world reports of AEs from a large number of populations, which may be overlooked in well‐designed clinical trials and have become a very valuable resource to support post‐marketing surveillance and early detection of drug safety issues. Thus, FAERS is a useful tool for identifying safety issues that may be associated with PCSK9i.^18^, ^19^, ^20^

**FIGURE 1:**
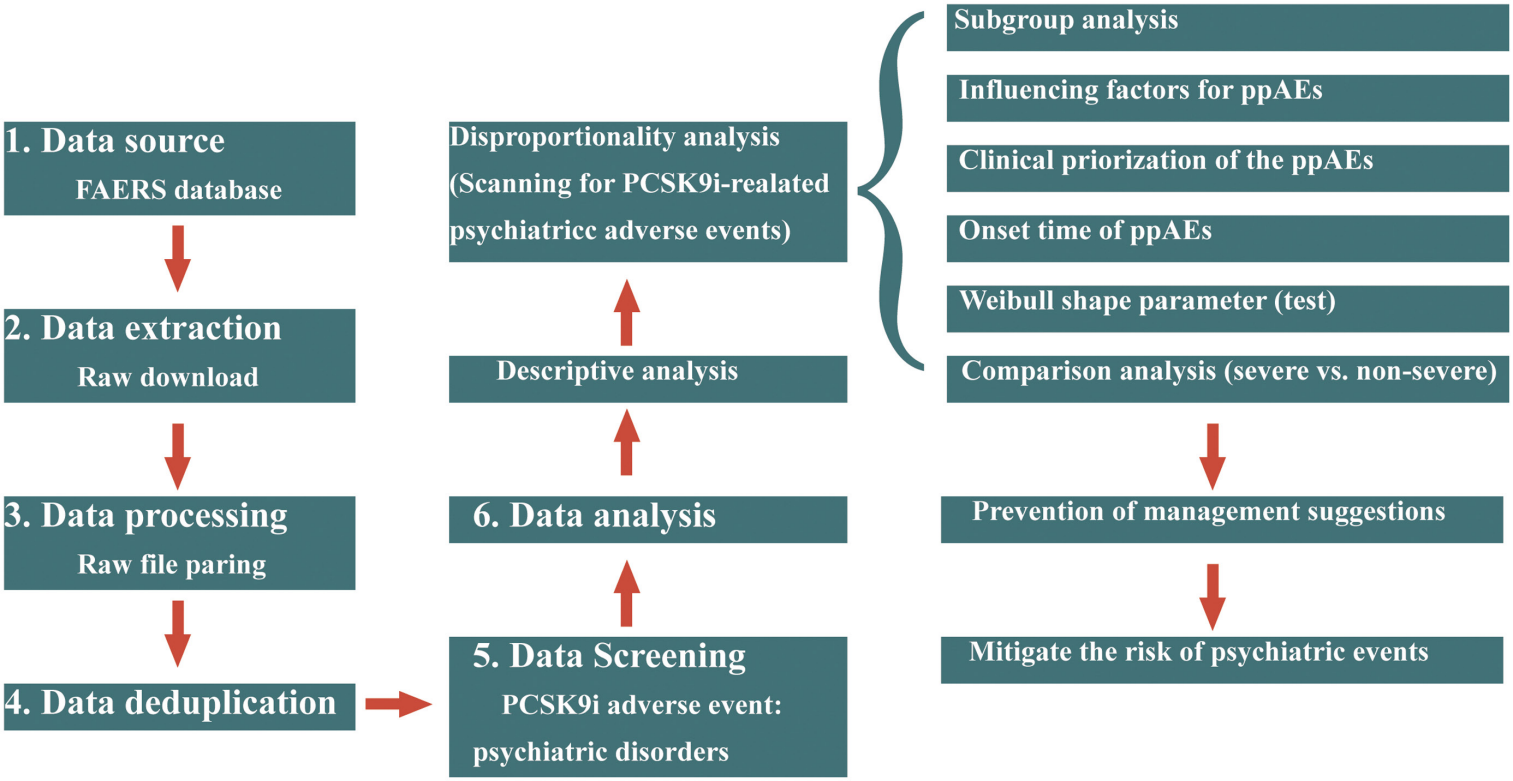
The process of data extraction, processing, and analysis from FAERS database.

This was a retrospective study with pharmacovigilance data extracted from the FAERS quarterly data extraction file (download link https://fis.fda.gov/extensions/FPD‐QDE‐FAERS/FPD‐QDE‐FAERS.html). We collected information on reports of PCSK9i treatment, including case number, AE, sex, age, country of reporting, outcome, duration of treatment, and date of AE. The design of this study is shown in Figure 1.

### 2.2 Data source

We conducted a retrospective search of PCSK9i related AEs reports between July 1, 2015 and March 31, 2023 in FAERS. Because there may be multiple versions of the same report, we identified and removed duplicates before statistical analysis to ensure the uniqueness of the reports.^21^

The earliest “FDA_DT” was deleted if “CASEID” was the same; if “CASEID” and “FDA_DT” were the same, the lower “PRIMARYID” was deleted. To clarify drug‐AE associations, we selected role_cod as the primary suspect (PS) and retained only reports from individuals aged 18 years and older. Comparisons of categorical variables were performed using Pearson's chi‐squared (*χ* ^2^) or Fisher's exact test, and comparisons of continuous variables, such as age and weight, were performed using the Mann–Whitney *U* test. Missing gender data were coded as “unknown”, and missing age data were estimated from the median of drug recipients in each group. All tests were two‐tailed, with *p* < 0.05 defined as significant. Data processing and statistical analysis were performed using Python and R software (version 4.3.0).

All PCSK9i related reports were extracted based on the generic name (drug name and prod_ai columns) and trade name (SEROQUEL in the drugname column) in the DRUG file (alirocumab, praluent, evolocumab, repatha, and pcsk9). Reports for atorvastatin and ezetimibe were also obtained.

To predict whether the incidence of AEs will increase or decrease over time, we performed a weibull shape parameter (WSP) test.^22^, ^23^ The weibull distribution is a probability distribution used to describe reliability and lifetime data, and the scale parameter *α* and shape parameter *β* are used to describe the weibull distribution. First, we calculate the median time to onset of AEs as follows: Time‐to‐onset (TTO) = EVENT time (EVENT_DT in DEMO file) ‐START time (START_DT in THER file). The results of the WSP test reflect three types of hazard models: the wear‐out failure type indicates that the hazard of AEs increases over time (*β* > 1, 95% CI >1); the early failure type indicates that the hazard of AEs decreases over time (*β* < 1, 95% CI <1), and the random failure type implies that the risk of AEs increases over time (*β* equal to or close to 1, 95% CI including values of 1). WSP tests were performed with Minitab statistical software (v20.0; Minitab LLC).

### 2.3 Signal mining

To quantify the association between PCSK9i and psychiatric AEs, we performed disproportionality analyses by calculating the ROR and the lower bound of the information component at a 95% credible interval (IC_025_). When the 95% CI lower bound of the ROR >1 and the IC_025_ > 0, it indicated that PCSK9i induced psychiatric AEs more frequently than other drugs.

First, AEs were coded using the preferred term (PT) according to the standardized Medical Dictionary for Regulatory Activities (MedDRA) at five levels: system organ class (SOC), high‐level group term (HLGT), high‐level term (HLT), preferred term (PT), and lowest level term (LLT).^24^ Based on the SOC (SOC: 10037175) = “Psychiatric disorders”, we obtained PTs for all psychiatric AEs in MedDRA (version 25.0) (*n* = 865, Table S1), ensuring that the PTs analyzed were from a clinical perspective. ROR and IC_025_ were calculated for all PTs according to the formula (Table S2).^25^ Next, the full PCSK9i reports were screened to minimize the impact of other AEs, concomitant medications, and indications for medications that may lead to psychiatric disorders.

In FAERS, the outcomes of AEs are categorized as serious or not serious. A report is defined as a serious medical event when it is documented in the OUTC file as a death, life‐threatening event, hospitalization, disability, or other serious medical event; the incidence of hospitalizations and serious medical events is calculated from the total number of psychiatric AEs.

### 2.4 Clinical prioritization of signals

AEs were semi‐quantitatively scored to determine clinical prioritization along five dimensions: number of reports, lower limit of ROR, frequency of death, characterization as a designated medical event (DMEs) (European Medicines Agency. Designated Medical Event (DME) list. 2016. https://www.ema.europa.eu/documents/other/designated‐medical‐event‐dme‐list_en.xls, accessed November 12, 2019) or important medical event (IMEs) (European Medicines Agency. Inclusion/exclusion criteria for the “Important Medical Events” list. https://www.ema.europa.eu/en/documents/other/eudravigilance‐inclusion/exclusion‐criteria‐important‐medical‐events‐list_en.pdf, accessed November 12, 2019), and biological plausibility (Table S3).^26^ AEs with composite scores of 0–4, 5–7, or 8–10 were identified as weak, moderate, or strong clinical priorities, respectively.

## 3 RESULTS

### 3.1 Descriptive analysis of psychiatric AEs in PCSK9i patients

**FIGURE 2:**
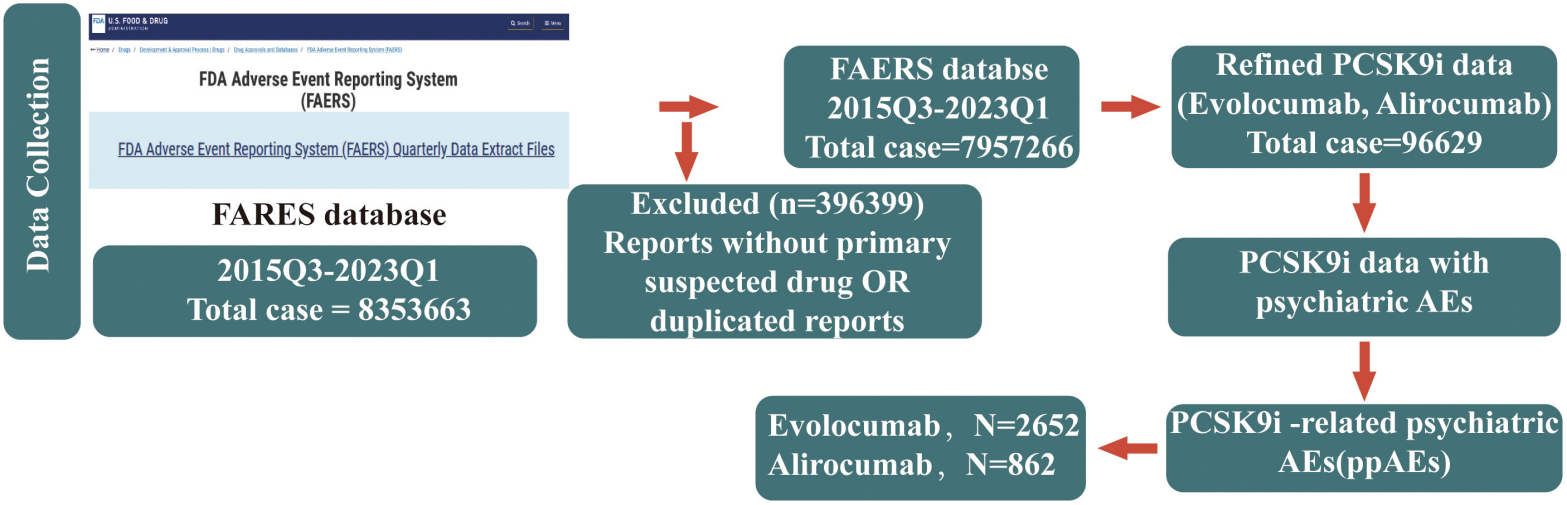
The flowchart includes data acquisition and data analysis.

**FIGURE 3:**
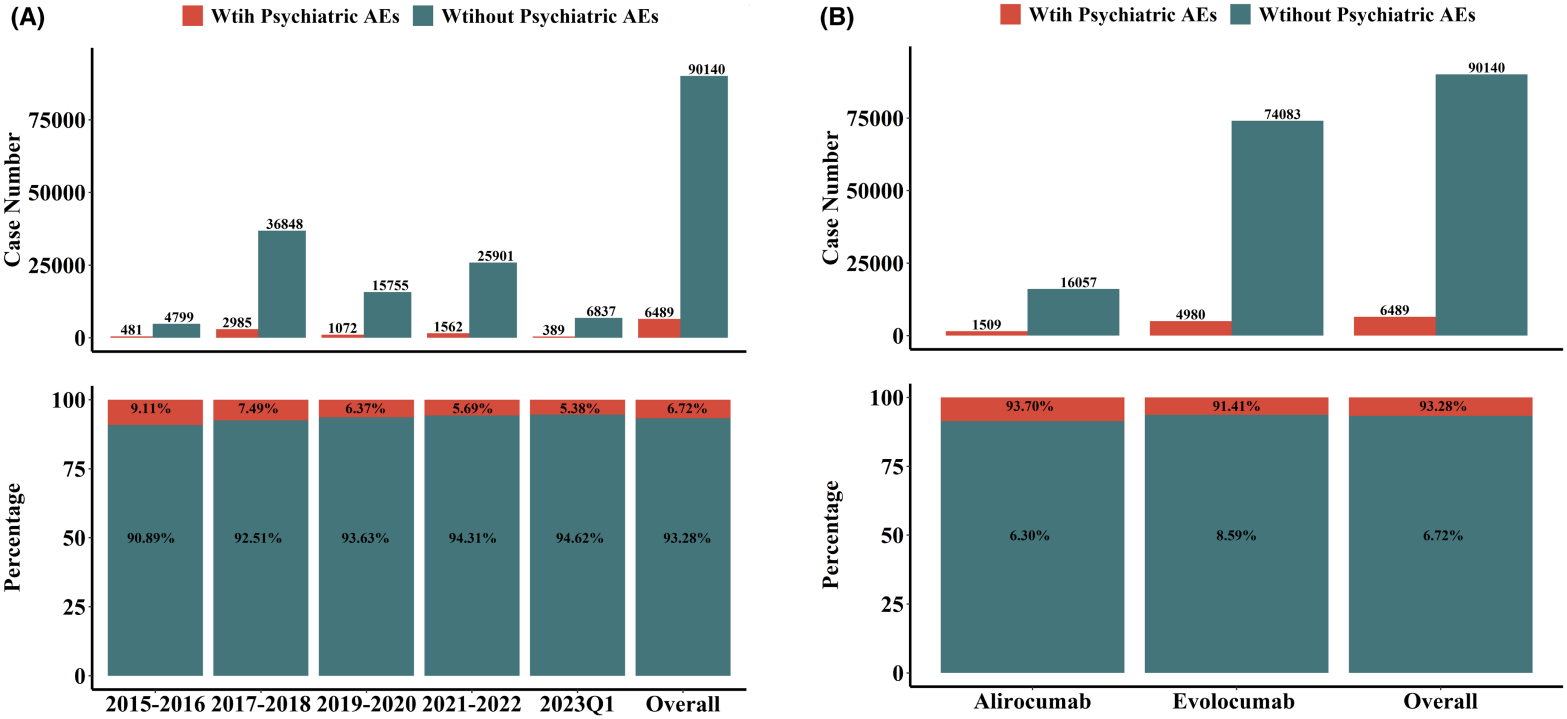
Statistics on the incidence of PCSK9i psychiatric AEs reported in FAERS during 2015–2023. (A) Calendar year data comparing the number of PCSK9i reports with psychiatric AEs to the number of PCSK9i reports without psychiatric AEs in FAERS during 2015–2023. (B) Overall comparison of the number of PCSK9i reports with psychiatric AEs versus PCSK9i reports without psychiatric AEs in FAERS, 2015–2023.

First, we investigated the occurrence of psychiatric AEs in patients treated with PCSK9i in FAERS from the third quarter of 2015 to the first quarter of 2023. The detailed data processing is shown in Figure 2. Over the 8‐year period, there were 7,957,266 reports in FAERS, and 96,629 reports were associated with the use of PCSK9i (79,063 and 17,566 for evolocumab and alirocumab, respectively). Of all PCSK9i reports, psychiatric AEs were reported in 6489 or 6.71% of the total number of cases (6489/96629, Figure 3A). In addition, the number of PCSK9i reports per two years represented a small fraction of the total number of cases, ranging from 5.38% to 9.11% (Figure 3B). The incidence of psychiatric AEs differed between the two PCSK9i; the number of reports was 4980 and 1509 for evolocumab and alirocumab, respectively, and the rate of reported psychiatric AEs was lower for evolocumab than for alirocumab (6.29% and 8.59%, respectively).

The median age of patients treated with both PCSK9i therapies was 68 years (evolocumab: interquartile range [IQR] 61–75, 1564 reports with age data; alirocumab: interquartile range [IQR] 62–74, 298 reports with age data). Using 65 years as the cutoff, we categorized patients into two distinct age groups, with the majority of patients treated with PCSK9i being older than 65 years, especially for alirocumab (*n* = 190, 63.76%). Among individual countries, the United States reported the highest number of psychiatric AEs with 86.56% (2313/2672). A higher proportion of female than male patients was reported in all reports (22.67% vs. 16.03%). Severe outcomes were observed in 41.40% (980/2367) of the cases. All cases were reported after the third quarter of 2015, and the number of reports increased annually from 2016 to 2019, with a slight decrease after 2020 (Table 1). Overall, psychiatric AEs under PCSK9i treatment strategies may represent a non‐negligible proportion of their associated AEs.

**TABLE 1:** Characteristics of PCSK9i‐related psychiatric AEs reports (July 1, 2015–March 31, 2023).

Characteristics	PCSK9‐related drugs induced Psychiatric AEs (*n* = 6489)	Evolocumab (*n* = 4980)	Alirocumab (*n* = 1509)
Gender, *n* (%)			
Female	1471	1238	233
Male	1040	821	219
Unknown		81	78
Age (years), *n* (%)			
≤ 65	768	660	108
>65	1094	904	190
Unknown		553	232
Age median (IQR)		68 (61–75)	68 (62–74)
Weight (kg), *n* (%)			
≤80	236	166	70
>80	153	122	31
Unknown		1854	429
Weight median (IQR)		78 (70–89)	77 (68–82)
Reported countries			
U.S.	2313	1912	401
Outside U.S.	359	230	129
Unknown		4	4
Outcome, *n* (%)			
Non‐serious	1387	1374	299
Serious cases	980	750	230
Death (DE)	8	1	7
Disability (DS)	34	28	6
Hospitalization—initial or prolonged (HO)	143	113	30
Life‐threatening (LT)	8	5	3
Other serious (important medical event) (OT)	787	603	184
Reporting year			
2023 Q1*	389	256	133
2021–2022	1562	1073	489
2019–2020	1072	727	345
2017–2018	2985	2555	430
2015–2016	481	369	112

### 3.2 Disproportionality analysis and screen for PCSK9i‐related psychiatric AEs (ppAEs)

**FIGURE 4:**
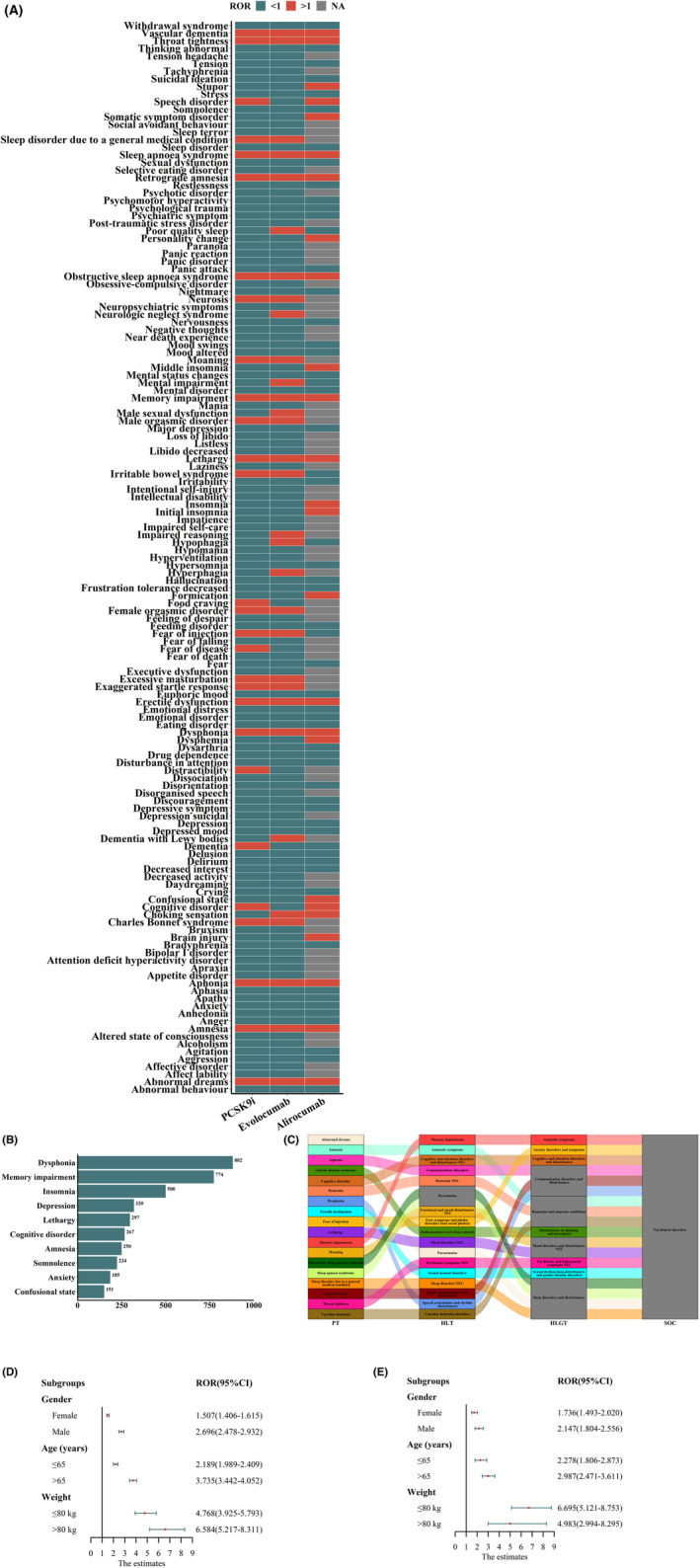
FAERS was scanned for ppAEs. (A) ROR of psychiatric AEs under the two PCKS9i treatment strategies (cases not less than 3). Red indicates a ROR value greater than 1, green indicates a ROR value less than 1, and gray indicates a ROR value that could not be calculated. (B) Number of reported cases of PCSK9i‐relared psychiatric AEs under the two PCKS9i treatment strategies. (C) Sankey diagram depicting the hierarchical relationship of PTs for ppAEs. PT denotes preferred terminology, HLT denotes high‐level terminology, HLGT denotes high‐level group terminology, and SOC denotes systemic organ class. (D–E) Forest plots showing the subgroup ROR of ppAEs under two PCKS9i treatment strategies.

We counted the categories and numbers of PCSK9i related psychiatric AEs. Overall, “dysphonia” (*n* = 882, 13.62%), “memory impairment” (*n* = 774, 11.96%), “lethargy” (*n* = 500, 7.72%), “cognitive disorder” (*n* = 320, 4.94%), and “amnesia” (*n* = 297, 4.59%) were the most frequently reported psychiatric AEs after PCSK9i treatment (Table 2). PTs (*n* ≥ 3) for all psychiatric AEs were analyzed for disproportionality using the complete FAERS as a comparator. After filtering for valid signals, we found that two PCSK9i were associated with different psychiatric AEs (Figure 4A). “Charles Bonnet Syndrome” had the lowest number of cases but high‐ROR signal intensity in the overall context (Figure 4A). Figure 4B illustrates the top 10 PTs in terms of number of reports.

**TABLE 2:** Number of different PTs among cases treated with PCSK9i during 2015–2023.

Psychiatric AEs (PT)	Number	Percentage (%)
Dysphonia	882	13.62
Memory impairment	774	11.96
Insomnia	500	7.72
Depression	320	4.94
Lethargy	297	4.59
Cognitive disorder	267	4.12
Amnesia	250	3.86
Somnolence	224	3.46
Anxiety	185	2.86
Confusional state	151	2.33
Fear of injection	151	2.33
Speech disorder	138	2.13
Sleep apnoea syndrome	129	1.99
Sleep disorder due to a general medical condition	124	1.92
Sleep disorder	108	1.67
Dementia	108	1.67
Throat tightness	98	1.51
Disturbance in attention	94	1.45
Obstructive sleep apnoea syndrome	82	1.27
Aphonia	78	1.20
Nervousness	76	1.17
Stress	63	0.97
Irritability	60	0.93
Depressed mood	58	0.90
Irritable bowel syndrome	56	0.86
Nightmare	54	0.83
Abnormal dreams	54	0.83
Erectile dysfunction	54	0.83
Hypersomnia	54	0.83
Mental disorder	54	0.83
Aphasia	42	0.65
Disorientation	40	0.62
Anger	40	0.62
Hallucination	38	0.59
Agitation	38	0.59
Middle insomnia	38	0.59
Emotional distress	38	0.59
Mental impairment	35	0.54
Hypophagia	32	0.49
Poor‐quality sleep	30	0.46
Fear	26	0.40
Dysarthria	24	0.37
Frustration tolerance decreased	22	0.34
Feeding disorder	21	0.32
Initial insomnia	18	0.28
Apathy	14	0.22
Mood swings	14	0.22
Major depression	14	0.22
Thinking abnormal	13	0.20
Formication	12	0.19
Mental status changes	12	0.19
Panic reaction	12	0.19
Psychomotor hyperactivity	12	0.19
Altered state of consciousness	12	0.19
Restlessness	11	0.17
Panic attack	11	0.17
Crying	11	0.17
Suicidal ideation	11	0.17
Eating disorder	10	0.15
Emotional disorder	10	0.15
Bradyphrenia	10	0.15
Paranoia	9	0.14
Food craving	9	0.14
Delirium	8	0.12
Anhedonia	8	0.12
Moaning	8	0.12
Mood altered	7	0.11
Personality change	7	0.11
Euphoric mood	7	0.11
Choking sensation	6	0.09
Brain injury	6	0.09
Abnormal behavior	6	0.09
Aggression	6	0.09
Decreased activity	6	0.09
Drug dependence	6	0.09
Withdrawal syndrome	6	0.09
Mania	6	0.09
Appetite disorder	5	0.08
Libido decreased	5	0.08
Fear of disease	5	0.08
Intentional self‐injury	5	0.08
Tension	4	0.06
Vascular dementia	4	0.06
Hyperphagia	4	0.06
Tension headache	4	0.06
Feeling of despair	4	0.06
Affect lability	4	0.06
Distractibility	4	0.06
Negative thoughts	4	0.06
Social avoidant behavior	4	0.06
Delusion	3	0.05
Somatic symptom disorder	3	0.05
Panic disorder	3	0.05
Charles Bonnet syndrome	3	0.05
Attention deficit hyperactivity disorder	3	0.05
Near death experience	3	0.05
Depressive symptom	2	0.03
Decreased interest	2	0.03
Psychiatric symptom	2	0.03
Retrograde amnesia	2	0.03
Stupor	2	0.03
Listless	2	0.03
Dysphemia	2	0.03
Discouragement	2	0.03
Obsessive‐compulsive disorder	2	0.03
Psychotic disorder	2	0.03
Exaggerated startle response	2	0.03
Neurosis	2	0.03
Male orgasmic disorder	2	0.03
Dementia with Lewy bodies	2	0.03
Selective eating disorder	2	0.03
Bipolar I disorder	2	0.03
Fear of death	2	0.03
Fear of death	2	0.03
Daydreaming	2	0.03
Laziness	2	0.03
Impaired self‐care	2	0.03
Depression suicidal	2	0.03
Sleep terror	2	0.03
Affective disorder	2	0.03
Loss of libido	2	0.02
Bruxism	1	0.02
Post‐traumatic stress disorder	1	0.02
Sexual dysfunction	1	0.02
Psychological trauma	1	0.02
Excessive masturbation	1	0.02
Female orgasmic disorder	1	0.02
Impaired reasoning	1	0.02
Executive dysfunction	1	0.02
Male sexual dysfunction	1	0.02
Neurologic neglect syndrome	1	0.02
Fear of falling	1	0.02
Apraxia	1	0.02
Impatience	1	0.02
Disorganized speech	1	0.02
Intellectual disability	1	0.02
Tachyphrenia	1	0.02
Neuropsychiatric symptoms	1	0.02
Hypomania	1	0.02
Dissociation	1	0.02
Alcoholism	1	0.02
Hyperventilation	1	0.02

Abbreviation: PT, preferred term.

Then, the 18 psychiatric disorder PTs highly associated with PCSK9i were then defined as a whole as PCSK9i‐related psychiatric AEs (ppAEs) (“dysphonia”, “memory impairment”, “lethargy”, “cognitive disorder”, “amnesia”, “fear of injection”, “speech disorder”, “sleep apnoea syndrome”, “sleep disorder due to a general medical condition”, “dementia”, “throat tightness”, “obstructive sleep apnoea syndrome”, “aphonia”, “abnormal dreams”, “erectile dysfunction”, “moaning”, “vascular dementia”, “charles bonnet syndrome”) (Table 3). Figure 4C provides the affiliation of the PT of ppAEs to other levels in MedDRA. A total of 3514 reports of ppAEs were captured after scanning the entire FAERS (evolocumab: 2652; alirocumab: 862). The ROR for ppAEs was again calculated using the full FAERS as the comparator (treating the 18 PCSK9i‐related psychiatric AEs described above as a class of AEs). The results showed that PCSK9i treatment was significantly associated with the occurrence of ppAEs (ROR = 2.54 [2.41–2.68]), but there was a difference in signal strength between the two drugs. Evolocumab treatment was more significantly associated with ppAEs, with a slightly higher ROR (ROR for evolocumab: 2.87 [2.61–3.81]; ROR for alirocumab: 2.79 [2.29–4.10]).

**TABLE 3:** Signal detection of PCSK9i associated psychiatric AEs.

PCSK9i						Evolocumab						Alirocumab					
Psychiatric adverse events (PT)	No. of cases, n (%)	ROR	Lower 95% CI	Upper 95% CI	IC_025_	Psychiatric adverse events (PT)	No. of cases, n (%)	ROR	Lower 95% CI	Upper 95% CI	IC_025_	Psychiatric adverse events (PT)	No. of cases, n (%)	ROR	Lower 95% CI	upper 95% CI	IC_025_
Dysphonia	882	6.16	5.87	6.47	2.62	Memory impairment	642	2.19	2.07	2.32	1.13	Dysphonia	264	10.14	9.28	11.08	3.32
Memory impairment	774	2.16	2.05	2.28	1.11	Dysphonia	618	5.28	4.98	5.59	2.39	Memory impairment	132	2.03	1.79	2.29	1.01
Lethargy	297	2.62	2.42	2.85	1.39	Lethargy	234	2.53	2.3	2.77	1.33	Obstructive sleep apnoea syndrome	132	253.37	217.43	295.25	7.02
Cognitive disorder	267	2.36	2.16	2.57	1.23	Amnesia	184	1.71	1.54	1.9	0.77	Insomnia	124	1.18	1.04	1.33	0.23
Amnesia	250	1.91	1.74	2.08	0.93	Fear of injection	149	9.9	8.78	11.16	3.26	Confusional state	96	1.58	1.37	1.83	0.66
Fear of injection	151	8.21	7.28	9.25	3	Obstructive sleep apnoea syndrome	135	57.57	49.5	66.97	5.57	Amnesia	66	2.77	2.33	3.29	1.45
Speech disorder	138	1.24	1.1	1.4	0.31	Aphonia	114	3.88	3.39	4.44	1.94	Sleep apnoea syndrome	63	6.52	5.45	7.79	2.64
Sleep apnoea syndrome	129	2.43	2.14	2.75	1.27	Throat tightness	84	1.95	1.67	2.28	0.96	Lethargy	63	3.06	2.56	3.66	1.59
Sleep disorder due to a general medical condition	124	3.14	2.76	3.57	1.64	Sleep apnoea syndrome	75	1.72	1.46	2.03	0.78	Speech disorder	36	1.78	1.41	2.25	0.82
Dementia	108	1.69	1.48	1.94	0.76	Sleep disorder due to a general medical condition	63	1.95	1.63	2.33	0.95	Cognitive disorder	32	1.55	1.21	1.99	0.62
Throat tightness	98	1.87	1.62	2.15	0.89	Mental impairment	52	1.22	1.01	1.49	0.29	Middle insomnia	16	2.08	1.47	2.96	1.01
Obstructive sleep apnoea syndrome	82	28.61	23.91	34.24	4.62	Vascular dementia	6	4.34	2.42	7.79	1.79	Erectile dysfunction	16	2.02	1.42	2.87	0.97
Aphonia	78	2.17	1.85	2.55	1.11	Moaning	4	2.1	1.03	4.25	0.9	Aphonia	15	2.3	1.6	3.3	1.14
Abnormal dreams	54	1.28	1.06	1.56	0.36							Abnormal dreams	14	1.83	1.26	2.67	0.83
Erectile dysfunction	54	1.24	1.02	1.5	0.31							Personality change	6	1.85	1.05	3.29	0.8
Moaning	8	3.43	2.07	5.69	1.59							Alcohol abuse	3	2.71	1.2	6.09	1.12
Vascular dementia	4	2.37	1.17	4.82	1.04							Vascular dementia	3	9.78	4.32	22.13	2.12
Charles Bonnet syndrome	3	19	7.75	46.59	2.41												

Next, to further explore the correlation between PCSK9i and ppAEs, stratified analyses were performed according to three aspects: age (≤65 and > 65 years), gender (female and male), and weight (≤80 kg and >80 kg). The results, shown in Table 4 and Figure 4D,E, discovered that the lower limit of the 95% CI of the ROR values was greater than 1 in all subgroups, indicating a strong statistical correlation between the two drugs and ppAEs. In female patients, the ROR values for the two drugs were 1.50 and 1.73, respectively, while in males they were 2.69 and 2.14, suggesting that males were more likely to develop ppAEs after PCSK9i treatment. In addition, most cases of ppAEs were more significantly associated with patients older than 65 years and weighing more than 80 kg.

**TABLE 4:** Subgroup analysis of PCSK9i‐related psychiatric AEs.

Drug	Psychiatric AEs	Total AEs	ROR	95% Lower	95% Higher
Evolocumab					
Female	627	1238	1.51	1.41	1.61
Male	433	821	2.70	2.48	2.93
Alirocumab					
Female	136	233	1.74	1.49	2.02
Male	92	219	2.15	1.80	2.56
PCSK9i					
Female	763	1471	1.54	1.45	1.64
Male	525	1040	2.58	2.39	2.78
Evolocumab					
≤ 65	323	660	2.19	1.99	2.41
>65	458	904	3.73	3.44	4.05
Alirocumab					
≤65	55	108	2.28	1.81	2.87
>65	77	190	2.99	2.47	3.61
PCSK9i					
≤65	378	768	2.20	2.01	2.41
>65	535	1094	3.60	3.34	3.89
Evolocumab					
≤80 kg	76	166	4.77	3.92	5.79
>80 kg	52	122	6.58	5.22	8.31
Alirocumab					
≤80 kg	45	70	6.70	5.12	8.75
>80 kg	10	31	4.98	2.99	8.30
PCSK9i					
≤80 kg	121	236	5.34	4.56	6.25
>80 kg	62	153	6.26	5.06	7.74

To assess the robustness of the signals, we performed two additional analyses. The first was to compare the signal strength of ppAEs with that of other lipid‐lowering drugs. PCSK9i use was more strongly associated with more frequent reports of ppAEs than statins [ROR = 3.59 (3.48–3.71)] and ezetimibe [ROR = 1.82 (1.74–1.90)] (Table 5); Second, according to E‐value calculations, uncontrolled confounders had a magnitude of 5.19 (4.53–5.94) to eliminate the association of evolocumab with ppAEs, and 5.02 (4.35–5.81) to eliminate the association of alirocumab with ppAEs.

**TABLE 5:** Reporting odds ratios of PCSK9i vs. the full database, atorvastatin, and ezetimibe as the comparator groups.

	PCSK9i	Comparator group	ROR	95% Lower	95% Higher
PCSK9i vs. Full database	3501/96,626	96,626/7,957,266	2.54	2.41	2.68
PCSK9i vs. Atorvastatin	3501/96,626	4387/435,749	3.59	3.48	3.71
PCSK9i vs. Ezetimibe	3501/96,626	1325/66,725	1.82	1.74	1.9

### 3.3 Analysis of concomitant events and factors influencing ppAEs

**FIGURE 5:**
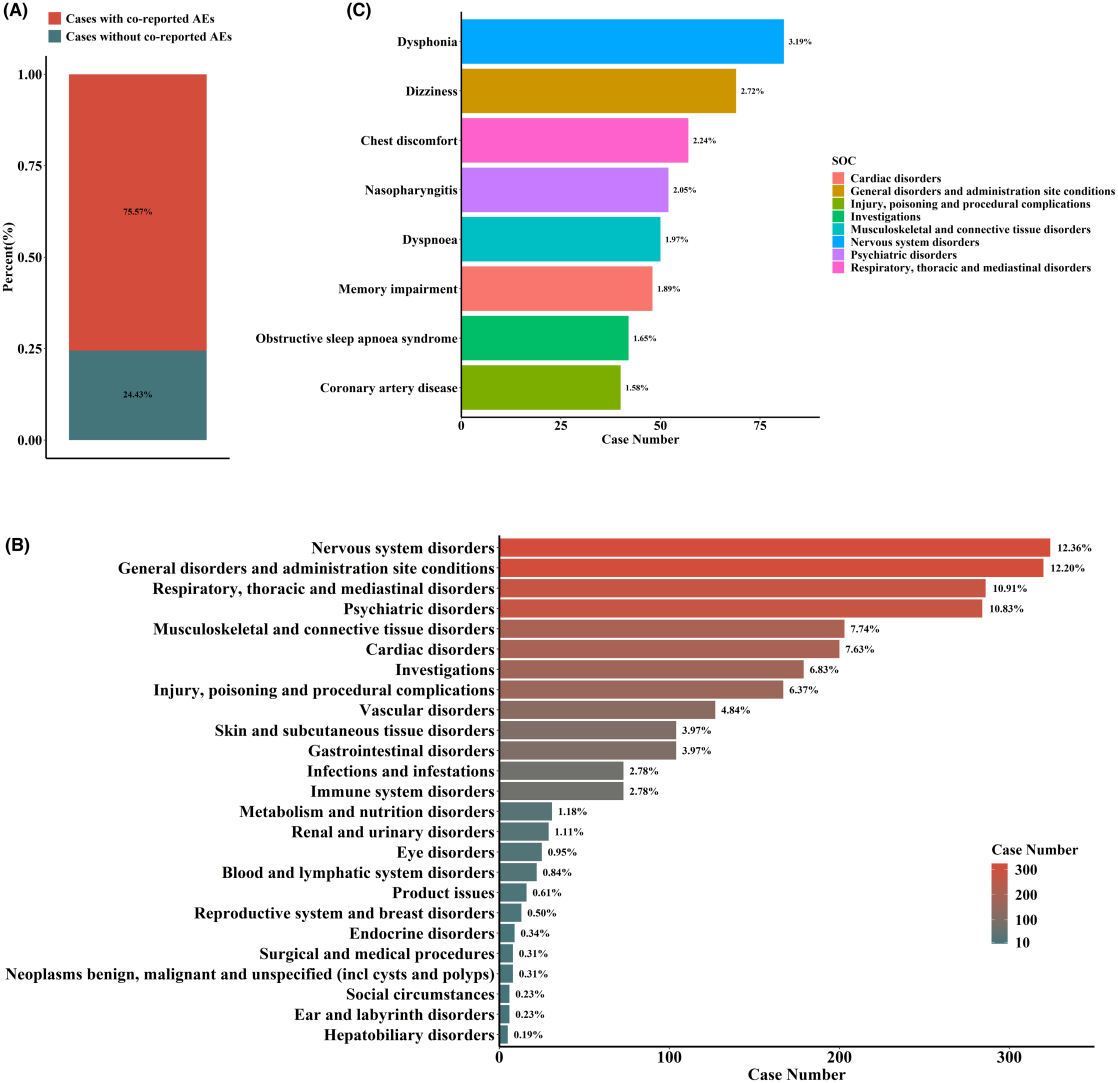
AEs co‐reported with ppAEs. (A) Percentage of ppAEs cases that did and did not present with co‐reported AEs. (B) Statistics on co‐reported adverse event PTs (SOC level). Percentage values labeled in the figure represent the proportion of cases with such adverse reactions among all ppAEs cases to the total number of cases with co‐reported adverse reactions. (C) Statistics on co‐reported adverse event PTs. Percentage values labeled in the graph represent the proportion of ppAEs cases in which such adverse reactions occurred.

Of the 3514 reports of ppAEs that we screened, 75.57% were accompanied by other AEs (Figure 5A). Among the concomitant AEs, the most frequently reported events were “nervous system disorders”, “general disorders and administration site conditions”, and “Respiratory, thoracic and mediastinal disorders” (top three), which were mentioned in more than 26.56% of reports. “Musculoskeletal and connective tissue disorders” were present in 7.74% of PCSK9i‐treated cases, whereas co‐reported “hepatobiliary disorders” were present in only 0.19% of cases (Figure 5B) (SOC level). In addition, “dysphonia” (3.19%), “dizziness” (2.72%), “chest discomfort” (2.24%), and “nasopharyngitis” (2.05%) associated with PCSK9i were in the top 10% of co‐reported AEs (Figure 5C) (PT level).

**FIGURE 6:**
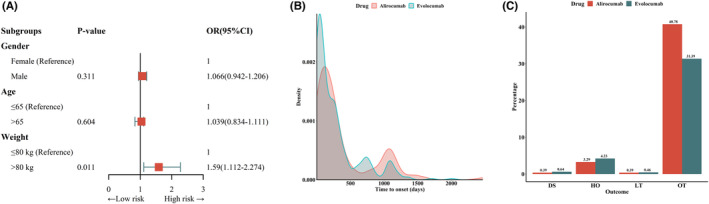
Results of univariate logistic regression analyses of factors influencing ppAEs (A), time to onset of ppAEs (B), and overall outcome indicators (C).

Univariate logistic regression analysis was used to examine factors that may influence the occurrence of ppAEs. Neither age (OR = 1.06 [0.94–0.94], *p* = 0.31) nor sex (OR = 1.03 [0.83–1.11], *p* = 0.60) significantly influenced the occurrence of ppAEs. Compared to patients weighing ≤80 kg, heavy patients (> 80 kg) were 1.59 times more likely to experience ppAEs (OR = 1.59 [1.11–2.27], *p* < 0.001) (Table 6, Figure 6A).

**TABLE 6:** Univariate logistic regression analysis of the odds ratio of ppAEs (controlling for multiple conditions).

Variable	Factor	OR	*p* Value	Lower limit of 95% CI	Uppper limit of 95% CI
Gender	Female (Reference)	1	–	–	–
	Male	1.06	0.31	0.94	1.20
Age	≤65 (Reference)	1	–	–	–
	>65	1.03	0.60	0.83	1.11
Weight	≤80 kg (Reference)	1	–	–	–
	>80 kg	**1.59**	**0.01**	**1.11**	**2.27**

*Note*: Bold values are statistically significant.

### 3.4 Clinical prioritization analysis of signals

In the above results, we scanned 18 ppAEs and six were categorized as IMEs, including “cognitive disorder”, “amnesia”, “sleep apnoea syndrome”, “dementia”, “erectile dysfunction”, and “vascular dementia” (Table 7). In addition, four psychiatric AEs were evaluated as strong clinical evidence of “++”. Based on the clinical priority assessment, 11 (61.11%), seven (38.89%), and zero psychiatric AEs were identified as weak, moderate, and strong levels of clinical priority, respectively. “Dysphonia” (*n* = 882, ROR = 6.84), “cognitive disorder” (*n* = 267, ROR = 2.62), and “amnesia” (*n* = 250, ROR = 2.12) were rated as moderate clinical priority with a maximum priority score of 6.

**TABLE 7:** Clinical priority assessing results of disproportionality signals.

PTs	*n*	ROR	Death (*n*)	IMEs–DMEs	Relevant evidence evaluation	Priority level (score)
Memory impairment	774	2.40	0	NA	++	Moderate (5)
Dysphonia	882	6.84	0	NA	++	Moderate (6)
Lethargy	297	2.91	0	NA	+	Weak (4)
Cognitive disorder	267	2.62	0	IME	++	Moderate (6)
Amnesia	250	2.12	0	IME	++	Moderate (6)
Fear of injection	151	9.11	0	NA	+	Moderate (5)
Speech disorder	138	1.38	0	NA	−	Weak2
Sleep apnoea syndrome	129	2.69	0	IME	+	Moderate (5)
Sleep disorder due to a general medical condition	124	3.48	0	NA	+	Weak4
Dementia	108	1.88	0	IME	+	Weak4
Throat tightness	98	2.07	0	NA	−	Weak3
Obstructive sleep apnoea syndrome	82	31.77	0	NA	+	Moderate (5)
Aphonia	78	2.41	0	NA	−	Weak3
Abnormal dreams	54	1.43	0	NA	−	Weak2
Erectile dysfunction	54	1.38	0	IME	−	Weak3
Moaning	8	3.81	0	NA	−	Weak2
Vascular dementia	4	2.63	0	IME	−	Weak2
Charles Bonnet syndrome	3	21.10	0	NA	−	Weak2

### 3.5 Time to onset analysis and weibull shape parameter (WSP) test

The time to ppAEs onset is shown in Figure 6B. Notably, 21.45% and 26.84% of patients reported the time to onset of ppAEs after treatment with evolocumab and alirocumab, respectively, with a median time to onset of 149 days (IQR 36–314 days) and 196 days (IQR 80–471 days), respectively. After WSP test, we found that the upper limit of the 95% CI for the shape parameter *β* was <1, suggesting that ppAEs are characterized by an early failure type. The results suggest a progressive decrease in the risk of ppAEs over time (Table 8).

**TABLE 8:** The results of time‐to‐onset analysis for signals with moderate prioritization.

PCSK9i	TTO (days)			Weibull distribution		Failure type
	Cases	–		Shape parameter		
	*n*	Median (IQR)	Min–max	*β*	95% CI	
Evolocumab	313	149 (36–314)	1–2008	0.74	0.67–0.80	Early failure
Alirocumab	119	196 (80–471)	1–2469	0.77	0.67–0.89	Early failure

### 3.6 Comparison of clinical characteristics of severe and non‐severe outcome

Figure 6C shows the outcome metrics of patients who received both PCSK9i treatments. Based on the different types of outcomes, we categorized the cases into severe and non‐severe outcomes and compared the clinical characteristics between the two groups. The results showed that after evolocumab treatment, the hospitalization rate and severe medical event rate of patients were lower than that of alirocumab [evolocumab: 385/1087 (35.41%); alirocumab: 114/255 (44.70%)].

Second, reports of severe and non‐severe outcomes were compared to identify risk factors (gender, age, weight) (Table 9). For gender alone, there was a statistically significant difference between severe and non‐severe cases treated with both PCSK9i [evolocumab: (*χ* ^2^ = 19.22, *p* < 0.0001), alirocumab: (*χ* ^2^ = 7.24, *p* = 0.007)]. “Obstructive sleep apnoea syndrome”, “dysphonia”, “sleep apnoea syndrome”, and “dementia” were more likely to be reported as severe outcomes. Notably, “dementia”, “obstructive sleep apnoea syndrome”, “moaning”, “vascular dementia”, and “charles bonnet syndrome” were reported as severe outcomes.

**TABLE 9:** Differences in clinical characteristics of severe and non‐severe reports.

	Serious cases (*n* = 401)	Non‐serious cases (*n* = 686)		*p*	Serious cases (*n* = 116)	Non‐serious cases (*n* = 139)		*p*
Age (years)	Evolocumab				Alirocumab			
Age median (IQR)	69 (61–75)	67 (61–75)			67 (60–73)	68 (61–75)		
≤65 (Reference)	120	197	0.26^a^	0.60^b^	13	39	0.01^a^	0.90^b^
>65	164	291			20	57		
Sex distribution								
Female, *n* (%)	189	432	19.22^c^	**<0.0001** ^d^	67	62	7.24^c^	**0.007** ^d^
Male, *n* (%)	184	237			31	61		
Weight (kg)								
Weight median (IQR)	77 (74–87)	86 (81–90)			77 (65–84)	79 (67–86)		
≤80 kg (Reference)	53	18	2.41^c^	0.12^d^	13	8	0.49^c^	0.48^d^
>80 kg	32	20			20	8		
Types of AEs (PT level), *n* (%)								
Memory impairment	242	238			36	82		
Dysphonia	258	210			201	57		
Lethargy	51	120			24	27		
Cognitive disorder	52	16			12	16		
Amnesia	110	26			20	38		
Fear of injection	10	90			0	2		
Speech disorder	27	33			18	15		
Sleep apnoea syndrome	69	0			54	6		
Sleep disorder due to a general medical condition	25	14			0	0		
Dementia	50	0			8	0		
Throat tightness	32	22			0	8		
Obstructive sleep apnoea syndrome	111	0			132	0		
Aphonia	24	45			6	6		
Abnormal dreams	14	16			2	10		
Erectile dysfunction	18	14			8	8		
Moaning	4	0			0	0		
Vascular dementia	6	0			3	0		
Charles Bonnet syndrome	2	0			0	0		

*Note*: *p* < 0.05 were considered statistically significant.

Bold values are statistically significant.

^a^ The chi‐square (*χ* ^2^) statistic of the Pearson chi‐square test.

^b^ Proportions were compared using Pearson chi‐square test.

^c^ The *Z* statistic of the Mann–Whitney *U* test.

^d^ The Mann–Whitney *U* test.

## 4 DISCUSSION

This pharmacovigilance study is based on real‐world data from FAERS and provides a comprehensive and systematic update of the latest evidence on PCSK9i‐related psychiatric safety. We identified 18 psychiatric AEs that were highly associated with PCSK9i treatment, highlighted the association between exposure to lipid‐lowering medications and psychiatric AEs, and explored the clinical characteristics of the occurrence of such AEs.

Psychiatric disorders are a stable but less common class of AEs associated with PCSK9i. PCSK9i was approved for marketing in 2015, and the number of psychiatric AE reports in that year was low, with a total of only 51 reports, 44 reports for evolocumab, and only 7 reports for alirocumab. Figure 3 shows that the number of PCSK9i AE reports per quarter increased significantly from 2015 to 2018, with the total number of reports in 2017–2018 being almost 7.5 times higher than in the previous 2 years. However, in the 2019–2020 window, the number of PCSK9i AE reports decreased significantly to only 42.22% of the previous number, and then gradually increased again in 2021–2022. We speculate that the decrease in the number of AE reports in 2019–2020 may be due to the global outbreak of COVID‐19 in 2020, where healthcare workers, patients, and drug R&D companies became more concerned about the impact of COVID‐19 on health and lives, and therefore neglected to report PCSK9i AEs. With the end of COVID‐19, the number of reported PCSK9i AEs was 7226 in the first quarter of 2023, based on which it is speculated that the number of PCSK9i reports may gradually stabilize from the first quarter of 2023.

FAERS‐based analyses showed that the proportion of psychiatric AEs was slightly higher in reports from patients treated with alirocumab than in reports from patients treated with evolocumab (6.30% vs. 8.59%). From the 96,629 reports of PCSKi‐related psychiatric AEs captured in FAERS, we identified 18 AEs that were strongly associated with PCSK9i, defining them as ppAEs. However, with the exception of “fear of injection”, none of the common psychiatric disorders such as “dysphonia”, “memory impairment”, “cognitive disorder”, “sleep apnoea syndrome”, and “obstructive sleep apnoea syndrome” that are listed in PCSK9i were mentioned in the drug labeling, suggesting that healthcare professionals need to pay further attention to unexpected AEs in the future.

The results of our analysis showed the highest number of dysphonia (*n* = 882) with a strong association with PCSK9i use (ROR = 6.84). Similar to our findings, Peter et al. reported dysphonia and paroxysmal loss of voice the day after the first subcutaneous injection of alirocumab (75 mg) in a 68‐year‐old female patient.^27^ However, none of the available literature on the target and mechanism studies of alirocumab explicitly identifies the larynx as a site of toxicity. We also found a significant association between PCSK9i use and obstructive sleep apnoea syndrome and sleep apnoea syndrome, especially obstructive sleep apnoea syndrome (ROR = 31.77, *n* = 82), which was the most common of the AEs with the PCSK9i with the highest association. Unexpectedly, however, there was no convincing clinical evidence of an association between PCSK9i and obstructive sleep apnoea syndrome. Obstructive sleep apnoea syndrome is characterized by recurrent collapse of the upper airway and neuromuscular dysfunction of the upper airway dilator muscles (especially the genioglossus) during sleep,^28^, ^29^ and is particularly susceptible to altered upper airway anatomy and defective compensatory neuromuscular activity.^30^ Diseases associated with neuromuscular injury also predispose to obstructive sleep apnoea syndrome, progressive respiratory muscle weakness and chronic hypercapnic respiratory failure.^31^ Since the pathogenesis of obstructive sleep apnoea syndrome is closely related to neuromuscular injury, and real‐world evidence suggests that the most common adverse effects associated with PCSK9i are muscle toxicity as well as myalgia.^32^ Therefore, we hypothesized that obstructive sleep apnoea syndrome after PCSK9i use may be a secondary effect of PCSK9i leading to myotoxicity, and that further studies are needed to investigate the effects of PCSK9i on obstructive sleep apnoea syndrome and the mechanisms underlying this potential association need to be further investigated.

We made innovative use of multidimensional analyses such as subgroup analysis, influencing factors, clinical prioritization of signals, and comparisons of serious and non‐serious outcomes to provide insight into the primary outcomes. Subgroup analyses showed that the frequency of reported ppAEs was significantly higher in women (*n* = 763) than in men (*n* = 525) for both PCSK9i, especially for evolocumab, where women accounted for 55% of the total number of psychiatric AE reports for PCSK9i, which is consistent with another study based on the Eudravigilance database.^7^ It may be due to estrogenic and pharmacokinetic changes that psychiatric disorders tend to occur in the female population, especially during the postpartum, menstrual, and menopausal periods.^33^ Elderly patients >65 years of age generally had higher ROR values than middle‐aged adults (2.20 vs. 3.60), suggesting that older adults taking PCSK9i may be more susceptible to psychiatric AEs. Another FAERS‐based study also reported that numerous AEs for evolocumab and alirocumab occurred more frequently in older adults >65 years of age,^32^ possibly due to age‐related decreases in both drug metabolism and excretory and digestive abilities, which may lead to increased plasma concentrations of PCSK9i.^34^ Therefore, caution should be exercised when prescribing PCSK9i to patients >65 years of age. Subgroup analyses were also performed based on body weight (≤80 kg and >80 kg), and we found that both categorized groups showed a significant ROR signal intensity greater than 1. Weiss et al. reported a significant increase in the likelihood of comorbid CNS disorders such as mood, eating, and sleep disorders in obese populations.^35^ We hypothesized that excess body weight may, to some extent, be an important factor in the induction of ppAEs by PCSK9i. However, when analyzed by univariate logistic regression, the sample sizes in the different groups were small, and conclusions regarding the factors influencing ppAEs need to be further validated by larger studies or clinical trials.

We also used a clinical prioritization assessment to prioritize ppAEs. The results showed that 7 moderate and 11 weak clinical priority signals were identified. The strongest priority signals were “dysphonia”, “cognitive disorder”, and “amnesia” with a score of 6. In addition, the strongest clinically prioritized psychiatric AEs were “dysphonia” (*n* = 138, 6 points), “memory impairment” (*n* = 774, 5 points), “cognitive disorder” (*n* = 267, 6 points), “amnesia” (*n* = 250, 6 points), “fear of injection” (*n* = 151, 5 points), “sleep apnoea syndrome” (*n* = 129, 5 points), and “obstructive sleep apnoea syndrome” (*n* = 82, 5 points), and these AEs, although expected and likely related to underlying psychiatric disorders.

TTO analysis showed a median time to ppAEs of 149 and 196 days for alirocumab and evolocumab, respectively, suggesting that the majority of patients will develop ppAEs some time after receiving PCSK9i therapy. All ppAEs had an early failure phenotype, suggesting a decreasing risk of ppAEs over time. Because PCSK9i is a long‐term persistent medication, ppAEs are likely to actually occur while patients are receiving PCSK9i therapy. Therefore, clinicians should be aware of the potential psychiatric toxicity of PCSK9i, especially when treating patients with large body weights (>80 kg), so that psychiatric AEs can be identified and mitigated in a timely and effective manner to help patients maintain long‐term medication.

Regarding the distribution of severity of outcomes, 59.27% of ppAEs were reported to be serious (slightly higher for alirocumab than for evolocumab, 56.69% vs. 65.58%).

In contrast, a study based on a hospital registry and nine pharmacovigilance databases reported that PCSK9i‐induced AEs were typically mild. The present analysis suggests that gender, but not age or weight, may be associated with an increased risk of ppAEs severity.^36^ Severe cases in the evolocumab group were older (median age 69 years vs. 67 years) and weighed less (median weight 77 kg vs. 86 kg) than non‐severe cases, but there was no statistical difference between the two groups. However, after both PCSK9i treatments, women had a significantly higher rate of severe ppAEs than men, with a statistically significant difference between the two groups, meaning that women were at higher risk of reporting severe ppAEs after PCSK9i treatment. We suggest that the higher incidence of serious outcomes in female patients may be related to the source of AE reporting. Women are more likely to suffer from anxiety, depression, and eating disorders due to the effects of estrogen, etc.^37^ FAERS is a self‐reporting system, and female patients may be motivated to report more serious AEs.

Clinicians are interested in whether their patients are more or less likely to safely benefit from PCSK9i therapy. Therefore, effective measures to prevent and manage these side effects are needed to improve the acceptability and effectiveness of treatment. First, a thorough assessment of the patient by the healthcare provider before prescribing, extra caution when using PCSK9i therapy in patients with pre‐existing language or memory disorders, patient education and instruction, and skilled nursing practice are also critical steps to mitigate AEs^38^, ^39^; Second, preventive measures can be taken for patients prior to the administration of the drug. If patients are anxious about injections, soft music can be played and the drug can be injected in a comfortable environment without overstimulation to alleviate the patient's mental stress; Third, clinicians need to fully consider the need for adjunctive medications. For example, sleep apnoea syndrome can be improved with sedative drugs.^39^ In the presence of psychiatric adverse reactions, the dose of PCSK9i may be appropriately reduced, although efficacy may be compromised as well as suspension or discontinuation of therapy due to serious adverse reactions.

While the primary responsibility of this study was to identify potential psychiatric hazards or risks associated with PCSK9i in an effort to minimize the risk of clinical medication events, this study has several limitations. First, the FAERS is a system based on spontaneous reports from healthcare professionals, patients, and insurance personnel with specific selective biases, such as different drug manufacturing lots, racial disparities in reported cases and inconsistencies in reporters' perceptions of specific adverse reactions, and the fact that not all reports of the occurrence of serious adverse reactions are collected. As a result, we were unable to establish causal relationships between ppAEs and their incidence; Second, more detailed information beyond the report of each case was not available. For example, for the COVID‐19 infections, we have not yet been able to determine whether COVID‐19 had an effect on the occurrence of ppAEs; Third, we were not able to fully distinguish between drug indications and the effect of other AEs on ppAEs. Although we analyzed other AEs associated with ppAEs, we only obtained the proportion of concomitant AEs and could not determine the effect of concomitant AEs on ppAEs; Fourth, we did not provide strong evidence of the underlying biological mechanisms of ppAEs.

In conclusion, this pharmacovigilance study comprehensively investigates and identifies 18 psychiatric AEs highly associated with PCSK9i and explores their clinical characteristics by performing disproportionality analyses based on real‐world data from the FAERS. The results of this study will help clinicians to recognize the different features of the psychiatric toxicity spectrum and to intervene early. As our study is exploratory, large‐scale prospective studies are needed for validation. In the future, we will consider clinical trials to further validate our findings.

## AUTHOR CONTRIBUTIONS

Wenqi Gao, Zhifang Deng, and Han Xiao were involved in the conceptualization and study design. Jue Liu, Hongjian Gong, and Xiaonan Cai were responsible for the collection, completeness, and accuracy of the data; Zhifang Deng wrote the draft of the article; Wenqi Gao revised the manuscript. All the authors were involved in drafting the article, analyzing and interpreting the data, and revising the article, and approved the final version.

## FUNDING INFORMATION

This work was supported in part by grants from the Top Medical Young Talents of Hubei Province (2019), and the Wuhan Yellow Crane Talent‐Outstanding Young Talents Program (Wenqi Gao, Zhifang Deng, and Hongjian Gong).

## CONFLICT OF INTEREST STATEMENT

None of the other authors reported a conflict of interest related to the study.

## Supporting information


Table S1

Table S2

Table S3

## Data Availability

The data that support the findings of this study are available in FDA Adverse Event Reporting System at https://urldefense.com/v3/__https://fis.fda.gov/extensions/FPD‐QDE‐FAERS/FPD‐QDE‐FAERS.html__;!!N11eV2iwtfs!u8T7AO36ZfpXmTu22lzGBqnnQOK20_Q6alNy1AuHXXfwqNYJy87YpAOAODiFUcPAuLBDrkD_Bo3KmYJl‐_JQAy0; https://fis.fda.gov/extensions/FPD‐QDE‐FAERS/FPD‐QDE‐FAERS.html.
