# Supplementary material for: Psychiatric disorders associated with PCSK9 inhibitors: A real‐world, pharmacovigilance study
Source: CNS Neurosci Ther. 2023 Nov 10;30(4):e14522. doi: 10.1111/cns.14522 (PMC11017405; doi:10.1111/cns.14522)
Supplement: Supplementary file 2 — Table S2 [file CNS-30-e14522-s002.doc]

Supplement Table 2. Calculation for the IC and ROR algorithms.

|  | Reports with target AE | Reports without target AE |
| --- | --- | --- |
| Reports with PCSK9i | a | b |
| Reports without PCSK9i | c | d |

Abbreviation:

a: the number of reports with suspect AEs of target drug;

b: the number of reports with other AEs of target drug;

c: the number of reports with suspect AEs of all other drug;

d: the number of reports with all other AEs of all other drug;

AE: Adverse event;

IC: Information component;

ROR: Reporting Odds Ratio.

The calculation formulas are shown below:

1. IC=log_2_^a(a+b+c+d)(a+c)(a+b)^
2. IC_025_=eIn(IC)-1.96(1/a+1/b+1/c+1/d)^0.5

(3) ROR=ad/b/c

(4) 95%CI=eln(ROR)±1.96(1/a+1/b+1/c+1/d)^0.5
