# Supplementary material for: Psychiatric disorders associated with PCSK9 inhibitors: A real‐world, pharmacovigilance study
Source: CNS Neurosci Ther. 2023 Nov 10;30(4):e14522. doi: 10.1111/cns.14522 (PMC11017405; doi:10.1111/cns.14522)
Supplement: Supplementary file 3 — Table S3 [file CNS-30-e14522-s003.doc]

**Supplement Table 3**. A rating scale assessing clinical priority of disproportionality signals.

| **Assessment items** | **2 points** | **1 point** | **0 point** |
| --- | --- | --- | --- |
| Number of target events | >50 | 10-50 | <10 |
| ROR | >5 | 2-5 | 1-2 |
| Mortality proportion | >50% | 25-50% | <25% |
| IMEs or DMEs | DME | IME | None |
| Current evidence evaluation | ++ | + | **-** |

Mortality proportion: percentage of cases in which death was reported as an outcome in the overall cases report for a particular adverse event. IMEs and DMEs are developed and updated by EMA (European Medicines Agency, 2020). ++ : AEs are mainly from the FDA Prescribing Information, the Summary of Product Characteristics of quetiapine posted by the MHRA, Phase 2/3 RCTs, or systematic reviews, with biological plausibility. + : AEs are mainly from other clinical trials, observational studies, or case reports/series with potential biological plausibility. - : AEs only emerging from disproportionality analyses. AEs, Adverse Events. DMEs, Designated Medical Events. IMEs, Important Medical Events. MHRA, Medicine and Healthcare Products Regulatory Agency. RCTs, Randomized Controlled Trials. ROR_025_, the lower limit of 95% confidence interval of ROR.
